# A kinetic model for Brain-Derived Neurotrophic Factor mediated spike timing-dependent LTP

**DOI:** 10.1371/journal.pcbi.1006975

**Published:** 2019-04-24

**Authors:** Sergio M. G. Solinas, Elke Edelmann, Volkmar Leßmann, Michele Migliore

**Affiliations:** 1 Institute of Biophysics, National Research Council, Palermo, Italy; 2 Institute of Neuroinformatics, University of Zurich and ETH Zurich, Zurich, Switzerland; 3 Institute of Physiology, Otto-von-Guericke-University, Medical Faculty, Magdeburg, Germany; 4 Center for Behavioral Brain Sciences (CBBS), Magdeburg, Germany; Northeastern University, UNITED STATES

## Abstract

Across the mammalian nervous system, neurotrophins control synaptic plasticity, neuromodulation, and neuronal growth. The neurotrophin Brain-Derived Neurotrophic Factor (BDNF) is known to promote structural and functional synaptic plasticity in the hippocampus, the cerebral cortex, and many other brain areas. In recent years, a wealth of data has been accumulated revealing the paramount importance of BDNF for neuronal function. BDNF signaling gives rise to multiple complex signaling pathways that mediate neuronal survival and differentiation during development, and formation of new memories. These different roles of BDNF for neuronal function have essential consequences if BDNF signaling in the brain is reduced. Thus, BDNF knock-out mice or mice that are deficient in BDNF receptor signaling via TrkB and p75 receptors show deficits in neuronal development, synaptic plasticity, and memory formation. Accordingly, BDNF signaling dysfunctions are associated with many neurological and neurodegenerative conditions including Alzheimer’s and Huntington’s disease. However, despite the widespread implications of BDNF-dependent signaling in synaptic plasticity in healthy and pathological conditions, the interplay of the involved different biochemical pathways at the synaptic level remained mostly unknown. In this paper, we investigated the role of BDNF/TrkB signaling in spike-timing dependent plasticity (STDP) in rodent hippocampus CA1 pyramidal cells, by implementing the first subcellular model of BDNF regulated, spike timing-dependent long-term potentiation (t-LTP). The model is based on previously published experimental findings on STDP and accounts for the observed magnitude, time course, stimulation pattern and BDNF-dependence of t-LTP. It allows interpreting the main experimental findings concerning specific biomolecular processes, and it can be expanded to take into account more detailed biochemical reactions. The results point out a few predictions on how to enhance LTP induction in such a way to rescue or improve cognitive functions under pathological conditions.

## Author summary

Storing memory traces in the brain is essential for learning and memory formation, and it occurs through synaptic plasticity processes. Timing-dependent Long-Term Potentiation (t-LTP) is a physiologically relevant type of synaptic plasticity that results from the repeated sequential firing of action potentials (APs) in pre- and postsynaptic neurons. T-LTP is observed during learning in vivo and is a cellular correlate of memory formation. T-LTP can be elicited by different patterns of combined pre- and postsynaptic activity that recruit distinct synaptic growth processes underlying t-LTP. The protein Brain-Derived Neurotrophic Factor (BDNF) is released at synapses and mediates synaptic plasticity in response to specific patterns of t-LTP stimulation in the theta frequency band, while other patterns mediate BDNF-independent t-LTP. Here, we developed a realistic computational model that accounts for our previously published experimental results of BDNF-independent 1:1 t-LTP (70 repeats of pairing 1 presynaptic with 1 postsynaptic AP) and BDNF-dependent 1:4 t-LTP (25 repeats of pairing 1 presynaptic with 4 postsynaptic APs). The model explains the magnitude and time course of both t-LTP forms and allows predicting t-LTP properties that result from altered BDNF turnover. Since BDNF levels are decreased in demented patients, understanding the function of BDNF in memory processes is important to counteract neurodegenerative diseases.

## Introduction

Brain-Derived Neurotrophic Factor (BDNF) is a member of the protein family of mammalian neurotrophins, further comprising nerve growth factor, neurotrophin 3 and neurotrophin 4/5. Neurotrophins are well known across the animal kingdom to support survival, ontogenetic development, differentiation, and stability of neurons in the entire nervous system[1,2]. In the mature nervous system, BDNF, in particular, serves additional roles by regulating functional and structural synaptic plasticity (reviewed e.g. in [1,3–5]).

In recent years, a wealth of data has been accumulated on the many roles of BDNF in regulating synaptic plasticity at glutamatergic and GABAergic synapses, e.g. in hippocampus, neocortex, amygdala, and cerebellum, unraveling signaling pathways of unprecedented complexity [1,6–10]. However, despite this well-established role of BDNF as a central activity-dependent mediator (i.e. switching on biochemical pathways that induce and maintain enhanced synaptic transmission [11–13]) and modulator (i.e., facilitating synaptic changes that are mediated by other signaling pathways [3,14–17]) of synaptic plasticity, the interplay between intra- and extracellular signaling pathways [18,19] that regulate and fine-tune BDNF-dependent synaptic changes is not well understood.

The overall picture is rather complex. BDNF consists of a protein homodimer that is generated exclusively in glutamatergic neurons from two identical peptide chains held together by noncovalent interactions. The precursor protein, pre-proBDNF, is sequestered into the endoplasmic reticulum, where the pre-sequence is cleaved off, yielding proBDNF. Intracellularly, proBDNF can be cleaved (by protein convertases, PCs and furin) into mature BDNF (mBDNF) and BDNF pro-peptide. All three BDNF species are thought to be assembled into secretory vesicles that are transported to the plasma membrane in soma, dendrites, and axons, where they release their content via Ca^2+^-dependent exocytosis [20].

Following secretion, remaining proBDNF can be cleaved by extracellular proteases (e.g. plasmin and matrix metalloproteinases). This is an important functional step since at this point it is determined whether mBDNF or proBDNF dependent signaling cascades are activated at a synapse. Because proBDNF and mBDNF activate signaling cascades that partially antagonize each other, the importance of knowing the exact identity of released BDNF can hardly be overestimated. While mBDNF preferentially binds to the tyrosine-kinase receptor B (TrkB) and, among other functions, supports LTP, proBDNF preferably docks to the p75 receptor, which mediates long-term depression (LTD) [8].

The complexity of BDNF control over neuronal growth, plasticity, and modulation, makes it difficult to carry out experimental studies to fully understand BDNF-dependent processes. Computational modeling can significantly help to untangle the interplay of these processes but, despite the widespread implications of BDNF signaling in structural and functional neuromodulation during normal and pathological physiological conditions, a biologically realistic model of how BDNF signaling instructs these changes is still missing. Except for a very recent example of a model of a positive BDNF feedback loop, to take into account experiments on inhibitory avoidance training [21], to the best of our knowledge there are no published models available that address BDNF-dependent pathways.

In this paper, we set out to investigate BDNF-dependent synaptic mechanisms, by implementing the first kinetic model of the central BDNF-dependent subcellular pathways underlying spike timing-dependent Long-Term Potentiation (t-LTP) at hippocampal synapses. For this purpose, we focused on TrkB-dependent processes at hippocampal Schaffer collateral to CA1 pyramidal cell synapses, for which extensive experimental work is available that can be used to constrain the parameter values [11,13]. We show that the model can capture the main experimental findings by using a minimal set of subcellular pathways, with which we can make specific predictions on how to enhance LTP induction in such a way to rescue or improve cognitive functions under pathological conditions.

## Results

### Experimental findings

**Fig 1:**
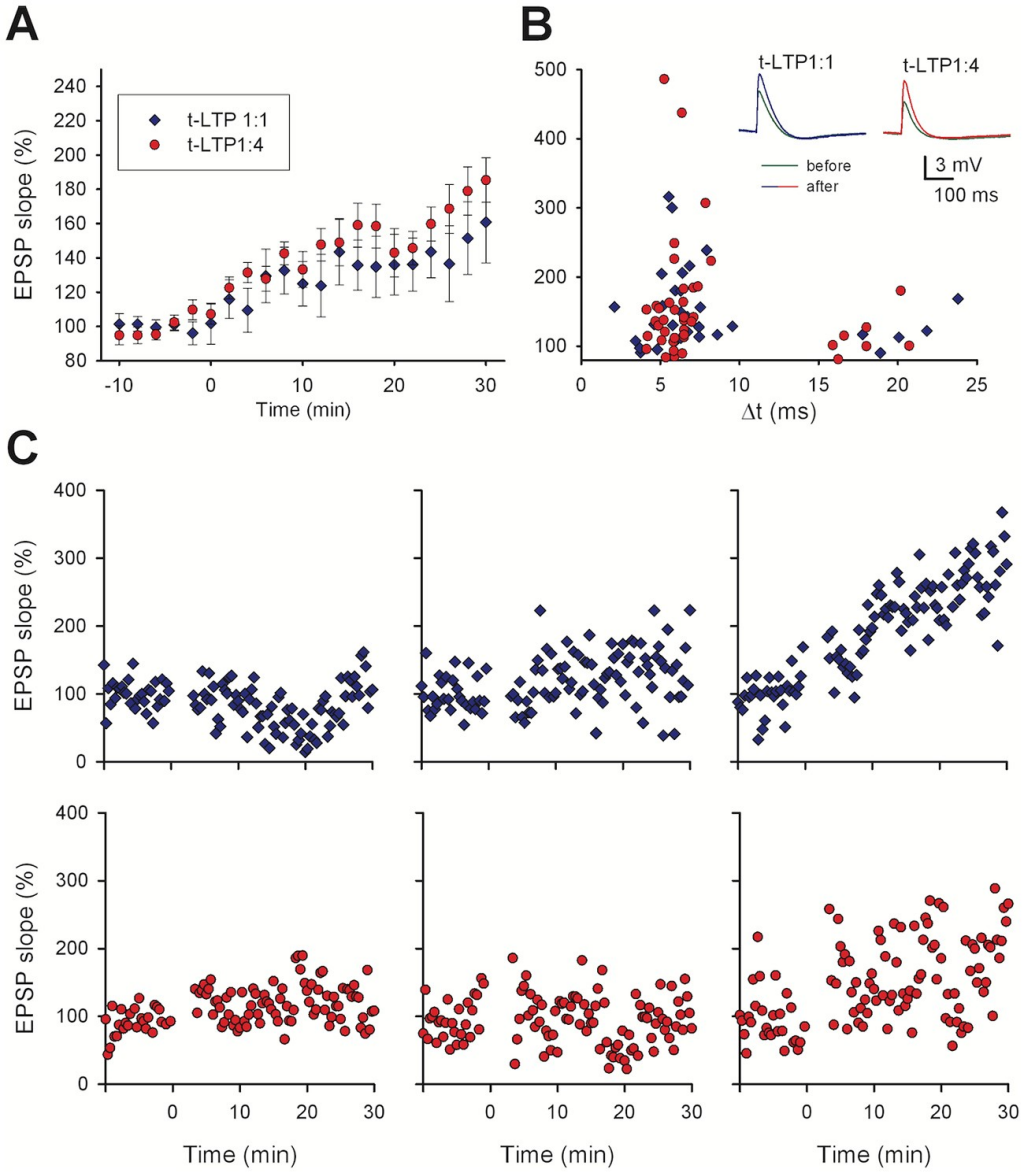
t-LTP expression in whole cell current clamp recordings. A) Average EPSP slope as a function of time (-10 to 0 min: baseline control). The 70x 1:1 t-LTP protocol (blue diamonds, n = 16) or the 25x 1:4 t-LTP protocol (red circles, n = 19) were executed at t = 0 min; in all cases average values (±sem) were calculated (bin width: 2 min), for cells with Δ*t* = 5 − 10 *ms*. B) Change of EPSP slopes in individual neurons 30 min after t-LTP induction for different Δ*t* between pre-synaptic activation and post-synaptic action potentials (70x 1:1 t-LTP n = 56, 25x 1:4 t-LTP n = 55; color coding as in panel A; data redrawn from Edelmann et al., 2015 (Fig 1B). The insets show typical somatic recordings of synaptic responses before (green) and after 1:1 t-LTP (blue) or 1:4 t-LTP (red). C) Typical examples of individual EPSP slope recordings following a conditioning period after a 10 min long baseline recording (upper panels paradigm 1:1, lower panels paradigm 1:4).

As a reference for our model, we considered the data from [11]. In the paper, the authors described t-LTP elicited in hippocampal pyramidal CA1 neurons by repeatedly pairing, with different delays (Δt), a single stimulation of the Schaffer Collaterals with one, two or four postsynaptic action potentials elicited at 200 Hz. These induction protocols are hereof designated 1:1, 1:2, and 1:4 t-LTP. The paper highlights important properties of the mechanisms underlying t-LTP. Their main results are summarized in Fig 1A, where the EPSP slope recorded in whole-cell patch clamp mode is plotted as a function of time. On average, the expression of t-LTP was relatively delayed, and it took approximately 30 min to reach its maximum expression (Fig 1A, data reproduced from Fig. 1B of [11]). The increase in synaptic strength after both 1:1 and 1:4 t-LTP was graded with time. Assuming that an individual synapse switches to a potentiated state following an all-or-none change [22,23], this progressive increase in the overall t-LTP observed at the soma suggests a distribution of transition times for different spines, driven by the time course of the processes underlying t-LTP induction and expression. For both protocols, a Δt>15 *ms* did not result in a significant t-LTP (Fig 1B), being consistent with other experimental findings [24,25]. As commonly expected, only short positive delays between pre- and postsynaptic stimulation are efficient to produce timing-dependent LTP, while longer delays reduce t-LTP magnitudes. In the experimental study that forms the basis of our model [11] a significant reduction of t-LTP was observed with positive time delays between 15–25 ms. It should be stressed that there was a rather large variability in the overall potentiation (i.e. in time course and magnitude) observed in recordings from individual cells, as demonstrated by the six typical cases of recording from different cells reported in Fig 1C. As will be discussed later, this finding is important for a better understanding of the interplay among the different processes underlying the induction of plasticity at each synaptic contact.

Additional properties of t-LTP are summarized in Table 1 and suggest that, in all cases, t-LTP induction was found to be postsynaptic and NMDA receptor-dependent. Instead, expression was found to be pre-synaptic for the 70x 1:1 protocol, post-synaptic for the 25x 1:4 protocol, and mixed for the 50x 1:2 protocol. The pre- or post-synaptic expression of t-LTP was experimentally determined by i) analyzing synaptic responses to short latency (50 ms) paired pulses inducing pre-synaptic short term plasticity (i.e. paired-pulse facilitation), ii) by infusing an inhibitor of AMPA receptor insertion into the postsynaptic membrane via the recording pipette solution, iii) by testing the AMPA/NMDAR current ratio, and iv) by using analysis of the coefficient of variation of EPSPs pre- vs. post LTP induction (see Fig.2 in [11]). E.g. pre-synaptic 1:1 t-LTP changes the glutamate release probability of release and therefore changes the temporal dynamics of short-term plasticity. Conversely, the post-synaptically expressed 1:4 t-LTP does not change short-term plasticity, but rather changes postsynaptic AMPA/NMDAR current ratio and depends on incorporation of new AMPA receptors into the postsynaptic membrane (all respective data shown in Fig.2 of [11]). Of note, the 1:1 t-LTP protocol was composed of 1 EPSP paired with 1 backpropagating action potential (bAP), whereas the 1:4 t-LTP protocol was composed of 1 EPSP paired with 4 (instead of 1) bAPs. Thus the 1:1 t-LTP protocol can be considered as being included (i.e. being a part of) in the 1:4 t-LTP protocol. One might thus expect that the mechanisms triggered by the 1:1 t-LTP protocol should also be activated by the 1:4 t-LTP protocol, but this was not experimentally observed [11].

**Table 1:** Summary of experimental results taken from Edelmann et al.[11].

Property	1:1 t-LTP	1:2 t-LTP	1:4 t-LTP
Location of t-LTP expression	Pre	Mixed	Post*
Location of t-LTP induction	Post	Post	Post
Number of repeated stimuli	70–100	50	25–35
Stimulus repetition frequency^¶^	0.5 Hz	0.5 Hz	0.5 Hz
Test frequency^§^	0.05 Hz	0.05 Hz	0.05 Hz
NMDA receptor-dependent	Yes	Yes	Yes
BDNF-dependent	No	Partial	Yes
Slow expression (30 min)	Yes	Yes	Yes
t-LTP Occlusion by 1:1 protocol	--	n.d.	No

n.d. = data not available.

* The paired pulse ratio (PPR) change induced by the 1:4 t-LTP protocol was small and not significant, but the PPR change distribution reveals occasional contamination by presynaptic expression.

^¶^ This frequency was used during t-LTP induction, each stimulus elicited either 1 EPSP paired with 1 bAP or 1 EPSP paired with 4 bAPs.

^§^ This frequency was used during the phase of t-LTP expression, each stimulus elicited a single EPSP.

Other experimental suggestions that could be used to further constrain the model implementation: (i) An increase in postsynaptic intracellular calcium, *[Ca*^*2+*^*]*_*i*_, was necessary to initiate a complex chain of biochemical reactions leading to the vesicular release of BDNF [3]; this process has stochastic dynamics that are ~10 times slower than glutamate release which resulted in a large variability of the time course of postsynaptic BDNF release with respect to the triggering event of a transient *[Ca*^2+^*]*_*i*_ elevation [26]. (ii) The postsynaptic BDNF release could last from a few seconds up to approximately 300 s ([20], supplementary Fig. 5 in [11]). (iii) There is no 1:1 (i.e. pre-synaptic) t-LTP expressed following 1:4 t-LTP stimulation [11]; this result may imply the existence of an additional mechanism, triggered by the 1:4 t-LTP protocol which is able to block the induction of 1:1 t-LTP. In summary, these experimental observations form a useful set of properties that give specific indications on what the model must be able to reproduce to be considered a reasonable representation of the many biochemical pathways that can be involved.

### The model

In agreement with experimental suggestions [22,23], the model was based on the assumption that any given individual synaptic contact, following the appropriate conditioning protocol, will change its state in an all-or-none manner. This was an important point to consider in comparing model and experimental findings since experimental recordings are customarily carried out from the soma, whereas the stimulation most likely involved an unknown number of synapses located in a relatively wide range of distances from the soma. The progressive increase in synaptic potentiation over time may thus be the result of an ensemble dynamics where different synapses undergo potentiation at different times. Unless explicitly stated otherwise, in discussing the model implementation we will always refer to individual synapses.

**Fig 2:**
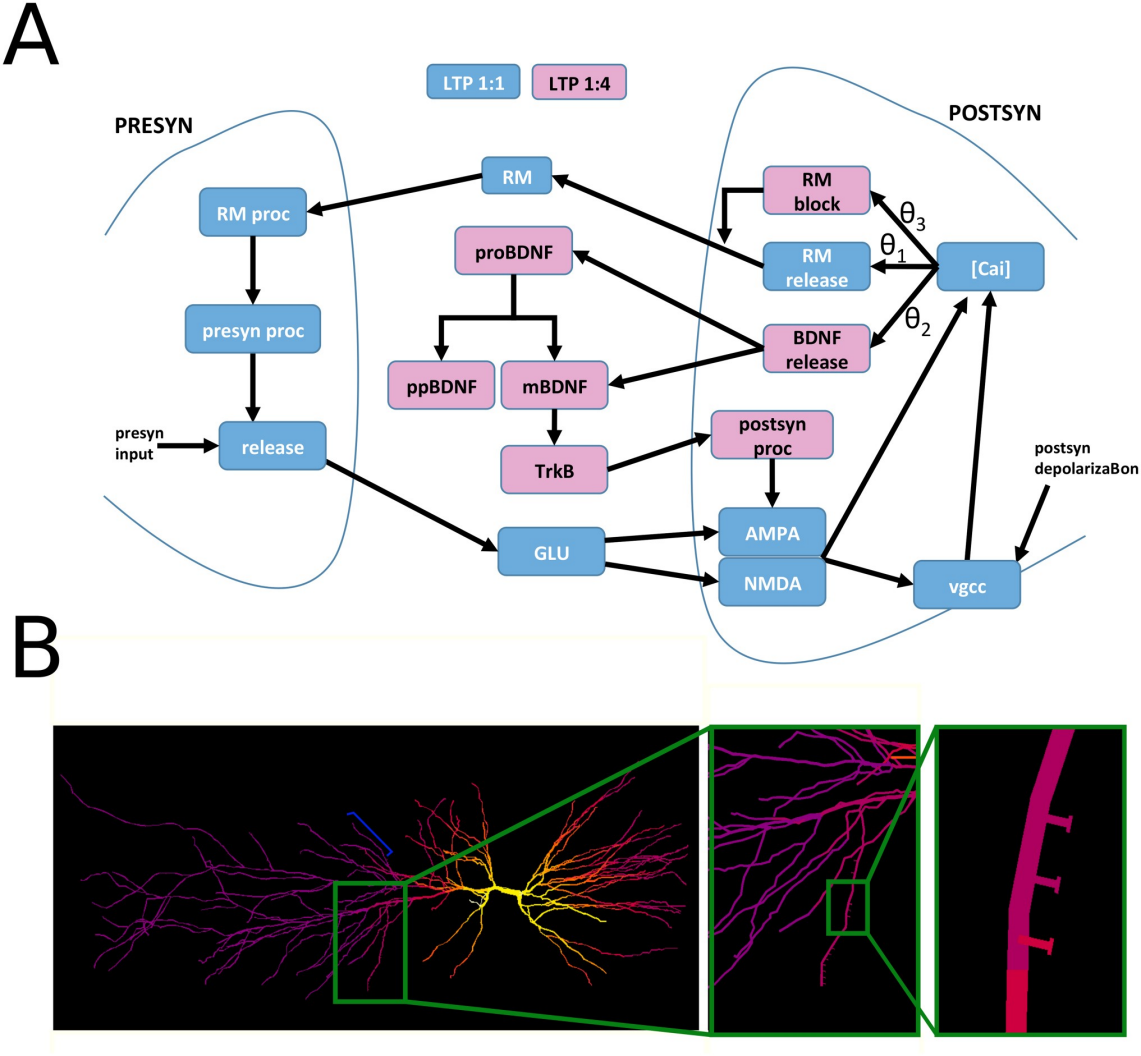
Schematic representation of our model for the BDNF-dependent t-LTP signaling cascade. A) For clarity, the biomolecular pathways involved with the 1:1 t-LTP or 1:4 t-LTP induction protocols are represented with blue and pink blocks, respectively. The model inputs are a synaptic activation ("presyn input", on the left) and a postsynaptic depolarization (on the right). The 70x 1:1 t-LTP cascade (blue) is activated if, during the train of stimuli, the [Ca^2+^]_i_ in the spine crosses the *θ*_1_ threshold frequently enough to allow RM to activate presynaptic RM-dependent processes (*RM proc*). This, in turn, will activate a pre-synaptic signaling cascade leading to the increase in glutamate release. The 25x 1:4 t-LTP cascade (pink) is activated by [Ca^2+^]_i_ transients above *θ*_2_, which trigger a delayed (0–300 s) fusion of vesicles containing mBDNF and proBDNF. The mBDNF activates the postsynaptic TrkB receptors that finally activate an intracellular, post-synaptic, signaling cascade leading to an increase of AMPA peak conductance; [Ca^2+^]_i_ transients above *θ*_3_, will result in a block of RM release. B) The CA1 model neuron used for all simulations, shown during the initial phase of an action potential backpropagating from the soma; the dendritic segments are color-coded for membrane potential (yellow represents a membrane potential above -20mV, purple represents -70mV). The insets show the location of 12 spines on a dendritic branch (branch38 of model morphology [30]). Six additional spines were placed on a different dendritic branch (branch8 of model morphology [30] indicated by the blue bracket on the left plot).

The biochemical pathways that we considered for this work are schematically represented in Fig 2A, and it is based on the hypothesis that distinct biochemical pathways are activated by different levels of intracellular [Ca^2+^] in the postsynaptic compartment [27,28]. In our model, there were three different [Ca^2+^]_i_ thresholds, θ_1_, θ_2_, and θ_3_. Ca^2+^ entry can independently occur through NMDA receptor or voltage-gated Ca^2+^ channels, both explicitly included in our model and known to drive postsynaptic BDNF secretion [29].

A transient [Ca^2+^]_i_ increase above each threshold activated one or many pathways in the spine head. The [Ca^2+^]_i_ range below θ_1_ corresponded to the non-plastic regime, i.e. any combination of pre- and/or post-synaptic input did not alter the current state of the pre- and/or post-synaptic mechanisms. Above θ_1_, it activated the 1:1 t-LTP signaling cascade (Fig 2A, blue boxes), which released a yet to be identified retrograde messenger (RM). This release activated presynaptic processes (*RM proc* and *presyn proc in* Fig 2A) resulting in a persistent increase in stimulus induced presynaptic glutamate release (*release*), in agreement with the change in the paired-pulse ratio observed experimentally [11]. Experimental findings suggest that neither nitric oxide (NO) [11] nor endocannabinoids were involved as RM. The fusion of postsynaptic BDNF vesicles was activated by a larger and more long-lasting Ca^2+^ transient ([Ca^2+^]_*i*_>θ_2_), which may be obtained with the 70x 1:4 t-LTP protocol (Fig 2A, dark pink blocks). The largest Ca^2+^ transients ([Ca^2+^]_*i*_>θ_3_) activated biochemical reactions blocking *RM* production. The rationale for this choice was that experimental recordings clearly show that the 1:4 t-LTP protocol did not induce presynaptic LTP [11]. For this to happen, there must be an activity-dependent (postsynaptic) process blocking the biochemical pathways leading to presynaptic LTP. In the model, we made the simple assumption that this process could be a Ca^2+^-dependent block of the retrograde messenger release, occurring for a [Ca^2+^]_i_ threshold (θ_3_) that is higher than the one for LTP induction. Other model behaviors were not affected by this assumption, and this scheme left open the possibility, for spines in which [Ca^2+^]_i_ reaches an intermediate concentration (θ_2_<[Ca^2+^]_*i*_< θ_3_), to account for a t-LTP with mixed pre- and post-synaptic mechanisms of expression that is obtained with a 1:2 t-LTP protocol [11].

The complete set of kinetic equations implementing the model (introduced in the next paragraph) were included into the membrane equation for each of the 18 explicitly modeled spines (see Methods). To roughly take into account the local dendritic temporal integration process, 12 of the 18 spines were distributed on one oblique dendrite (Fig 2B), whereas the remaining 6 spines were distributed on a different dendrite (see blue bracket in Fig 2B) and had different values for the θ_i_ (see Table 2).

**Table 2:** Model parameters.

Parameter	Model Value	
*α*_*fuse*_	5.5e-7 ms^-1^	
*α*_*RM*_	0.007 ms^-1^	
*σ*_1_	0.01e-3 mM	
*σ*_3_	0.0001 mM	
*α*_*CRM*_	1e-3 mM/ms	
*α*_*RMp*_	1e-3 ms^-1^	
*θ*_*RM*_	0.02 mM	
*σ*_*RM*_	0.001 mM	
*RM*_*inf*_	0 mM	
*α*_*pp*_	5.5e^-7^–16.5e^-7^ *ms*^-1^	
*α*_*RMpU*_	0.54	
*θ*_*U*_	0.15 mM	
*σ*_*U*_	0.001 mM	
[*Ca*]_*i*_*max*_	0.16 mM	
*v_PC*	0.002 mM	
*v_BDNF*	0.002 mM	
*α*_*PC*_	1e-4 mM/ms	
*α*_*diff*_	0.01 μM/ms	
*θ*_*TrkB*_	0.0002 mM	
*σ*_*TrkB*_	0.00001 mM	
*α*_*post*_	5.5e-6 ms^-1^	
*α*_*AMPA*_	1.5	
*θ*_*AMPA*_	0.01 mM	
*σ*_*AMPA*_	0.00001 mM	
Parameter	Branch38	Branch8
*θ*_1_	0.046 mM	0.004 mM
*θ*_2_	0.1 mM	0.045 mM
*θ*_3_	0.12 mM	0.052 mM

### The model: Presynaptic mechanisms

The presynaptic mechanisms specific for this work were added to the phenomenological model discussed in [31], and described by the following set of equations:

$$\frac{dx}{dt}=\frac{z}{\tau_{rec}}-U_{SE}\cdot x\cdot \delta (t-t_{spike})$$ (1)

$$\frac{dy}{dt}=-\frac{y}{\tau_{in}}+U_{SE}\cdot x\cdot \delta (t-t_{spike})$$ (2)

z=1−x−y (3)

where *δ*(*t*) is a delta function, *t*_*spike*_ was the time of arrival of a spike at the pre-synaptic terminal, the variables *x*, *y*, and *z* are the fraction of resources in the recovered, active, and inactive states, respectively, and *U*_*SE*_ was proportional to the glutamate released by each synaptic stimulation. They reproduced the stereotypical synaptic response dynamics between pyramidal neurons under physiological conditions. The values for the presynaptic parameters were those used in Ref. [31], with *U*_*SE0*_ = 0.1, *τ*_*rec*_ = 0.8 sec, and *τ*_*in*_ = 3 ms. This presynaptic mechanism has been previously shown to reproduce experimental findings on the normalization of temporal summation of synaptic inputs targeting distal or proximal dendrites of CA1 pyramidal neurons [32].

*U*_*SE*_ was additionally modulated by retrograde messenger-dependent pathways described by the following equations:

$$\begin{array}{l} \frac{dRM}{dt}=\alpha_{RM}\cdot (RM-RM_{\text{inf}})+\alpha_{CRM}\cdot S(cai,\theta_{1},\sigma_{1})\cdot [1-S(cai,\theta_{3},\sigma_{3})]\\ -\alpha_{RMp}\cdot (RM-RM_{\text{inf}})\cdot S(cai,\theta_{RM},\sigma_{RM}) \end{array}$$ (4)

$$\frac{dRMp}{dt}=\alpha_{RMp}\cdot (RM-RM_{\text{inf}})\cdot S(cai,\theta_{RM},\sigma_{RM})-\alpha_{pp}\cdot RMp$$ (5)

$$\frac{dpp}{dt}=\alpha_{pp}\cdot RMp$$ (6)

$$U_{SE}=U_{SE0}\cdot (1+\alpha_{RMpU}\cdot S(pp,\theta_{U},\sigma_{U})),$$ (7)

where RMp and pp were presynaptic processes activated in cascade by RM accumulation in the synaptic cleft (“RM proc” and “presyn proc” in Fig 2A), and $S(i,j,k)=\frac{1}{1+e^{\left(j-i\right)/k}}$ is the typical sigmoidal logistic function ubiquitously observed in biological systems [33]. Our hypothesis is that the activation of these mechanisms follows a dose-response curve. These processes are usually implemented with a sigmoid or a Hill function. Although the latter can be more easily related to the biomolecular pathways it represents, it also implies a significantly higher computational cost (for NEURON running on a PC we verified a 35% difference in CPU time). This occurs because of the internal representation of the computational algorithms used to calculate an *exp* (in a sigmoid function) or a *power* (in the Hill function) on any given computer. Since we plan to use this model on a large-scale network, we have preferred to implement these curves with a sigmoid function. The *cai* was the intracellular calcium concentration [Ca^2+^]_i_. Note that *pp* does not have a decay term. This ensures that a potentiated synapse does not spontaneously fall back to its non-potentiated state. It is technically possible to continue to present the induction protocol for infinite time yielding to RM release and consequent infinite growth of *pp*. However, this exploratory modeling work does not consider this remote possibility.

### The model: Post-synaptic mechanisms

Post-synaptic mechanisms are activated by different levels of [Ca^2+^]_i_, with the instantaneous Ca^2+^ dynamics determined by the complex interaction between AMPAR and NMDAR conductances, voltage-gated Ca^2+^ channels, and all other active and passive membrane properties. All the equations regulating the instantaneous Ca^2+^ dynamics were taken from a previously published CA1 neuron model [30] (ModelDB a. n. 55035). We reported here only the equations of the new mechanisms introduced in this work, and directly related to the synaptic transmission pathways using [Ca^2+^]_i_ as an input (see Methods for detail on how to access the full model).

The fusion of BDNF-containing vesicle with the spine head membrane is a complex process, possibly involving several biochemical pathways for which there are not enough experimental constraints to build a detailed kinetic scheme. For this reason, we implemented the effective action of these pathways using two mechanisms, accounting for the dependency of BDNF vesicle fusion probability and delay with respect to the STDP induction protocol.

The first mechanism is implemented with an empirical variable, which we called *intracellular signaling* (*is*). It is based on the experimental findings [11] suggesting that the fusion of BDNF-containing vesicles occurs only for conditioning protocols consisting of at least 25 induction stimuli repeated at a frequency close to 0.5 *Hz*, while no fusion was achieved in response to test stimulations at 0.05 Hz. In the model, this was obtained by increasing *is* by a fixed amount every time [Ca^2+^]_i_ crossed the θ_2_ threshold, and decreasing it with a time constant of 8 *s*. The fusion was allowed to occur only for *is*>0.15.

With the second mechanism, we took into account the experimental findings ([20], supplementary Fig. 5 in [11]) showing that the fusion of an individual vesicle containing BDNF, when activated, is a stochastic process occurring over a relatively long time window. We modeled all the involved processes by assuming that the fusion process happened with probability *pf* (defined for [Ca^2+^]_*i*_>θ_2_), and delay *df*, calculated as:

$$pf=\{\begin{array}{cc} \frac{{\left[Ca\right]}_{i}-\theta_{2}}{{\left[Ca\right]}_{i\_\text{max}}-\theta_{2}} & \left(is>0.15\right)\\ 0 & \left(is\leq 0.15\right) \end{array}$$ (8)

$$df=300\cdot (1-\frac{{\left[Ca\right]}_{i}-\theta_{2}}{{\left[Ca\right]}_{i\_\text{max}}-\theta_{2}})\cdot rand[0,1],$$ (9)

and assuming that

Fused_vescicles=f(pf,df) (10)

The function *f(pf*,*df)* keeps track of how many vesicles, in each synapse, have fused with the plasmatic membrane and were in the process of releasing BDNF. For each synapse, this function is increased by 1 with probability *pf* after a time interval *df* from the instant at which [Ca^2+^]_i_ crosses the *θ*_*2*_ threshold. The function is updated, asynchronously for each vesicle, every 1 *ms* of simulated time, theoretically leading to a minimum interval of 1 ms between the start of a new vesicular release, with a maximum number of available vesicles in each synapse set at 200, consistent with experimental data [34]. The function decreases by 1 (with a minimum value of zero) every time a vesicle has been fused for 30min. Random numbers from a uniform distribution in the interval [0–1] were used to choose the values for *df*, and *pf*; it should be stressed that this choice should not be considered as parametric randomization but, rather, as a way to introduce into the model an intrinsic stochastic behaviour.

During the time the [Ca^2+^]_i_ remains above the *θ*_*2*_ threshold the process leading to the release of a quantal amount of BDNF is active. In this time window, the fusion process of individual vesicles is initiated with probability *pf* and results in an actual fusion starting at a random time *df* (up to 300 sec) from activation, in agreement with experimental observations ([20], supplementary Fig. 5 in [11]). This also means that for [Ca^2+^]_i_ remaining for a prolonged time above threshold, more fusion processes are started. Once a vesicle has fused with the membrane, it continuously releases a fraction of the stored mBDNF and proBDNF for some time. The experimental evidence for this process is indirect, and it suggests a lower and an upper bound for the overall process: the release lasts for at least 5 min [29], but the overall LTP induction proceeds for approximately 30 min [11]. We made the somewhat simplifying and minimal assumption that the BDNF release lasts for 30 min. However, if this assumption would be invalidated by new experimental data, for example with longer experimental recordings of the BDNF release from single vesicles, the model could be straightforwardly revised by including an additional variable activated by a short BDNF release and slowly decaying over a period of 30 min. In any case, it is important to stress that in order to be consistent with the available experimental findings, the process modulating the magnitude of induced LTP must have a time course of approximately 30 min.

The ratio between mBDNF and proBDNF inside these vesicles is unknown. Indirect experimental evidence [35,36] indicate for the mBDNF:proBDNF proportion a value in the range of 10% to 90%. This ratio also depends on the pH inside the vesicle [20,37]. Since in our mouse brain slices we detected ~66% mBDNF vs. ~33% proBDNF in cell lysates, we used a 70%:30% proportion of mBDNF and proBDNF, respectively. In the Golgi apparatus and in BDNF-containing vesicles proBDNF can be cleaved by protein convertases (PC) into mBDNF and BDNF pro-peptide. Following the release, remaining proBDNF can be cleaved by plasmin or matrixmetallo proteinases [20]. To empirically model extrasynaptic diffusion and reuptake [38,39], mBDNF, proBDNF, and PC were all assumed to decay at a constant rate α_diff_. The overall level of mBDNF present in the synaptic cleft determined the extent of TrkB receptor activation, Through a chain of postsynaptic processes represented by *postsyn* in Fig 2A, TrkB induces t-LTP by increasing the AMPA receptor conductance [11]. We implemented these processes as:

$$\frac{dproBDNF}{dt}=\alpha_{fuse}\cdot 0.3\cdot Fused\_vesicles\cdot v\_BDNF-\alpha_{PC}\cdot PC\cdot proBDNF-\alpha_{diff}\cdot proBDNF$$ (11)

$$\frac{dmBDNF}{dt}=\alpha_{fuse}\cdot 0.7\cdot Fused\_vesicles\cdot v\_BDNF+\alpha_{PC}\cdot PC\cdot proBDNF-\alpha_{diff}\cdot mBDNF$$ (12)

$$\frac{dPC}{dt}=\alpha_{fuse}\cdot Fused\_vesicles\cdot v\_PC-\alpha_{diff}\cdot PC$$ (13)

$$TrkB=mBDNF\cdot S(mBDNF,\theta_{TrkB},\sigma_{TrkB})$$ (14)

$$\frac{dpost}{dt}=\alpha_{post}\cdot TrkB$$ (15)

$$g_{AMPA}=g_{\text{max}}\cdot [1+\alpha_{AMPA}\cdot S(post,\theta_{AMPA},\sigma_{AMPA})],$$ (16)

where *g*_*AMPA*_ is the peak AMPA conductance, *g*_*max*_ its maximum value before LTP, and *post* represents the long-term effects of TrkB-dependent processes on the overall AMPA conductance. Note that *post* does not have a decay term. This ensures that a potentiated synapse does not spontaneously fall back to its non-potentiated state. It is technically possible to continue to present the induction protocol for infinite time yielding to BDNF release and consequent infinite growth of *post*. However, this exploratory modeling work does not consider this remote possibility.

The overall model was too complex to attempt an automatic fitting procedure, especially considering that there were not enough clear experimental constraints to reduce the number of free parameters. For this reason, the parameters were set in two steps: 1) for each block shown in Fig 2A, an initial estimate for the involved parameters was obtained by presenting inputs that mimic the signals that could be generated in the full model, and manually adjusting the values to obtain what we considered a reasonable output signal; 2) test simulations of the full model were carried out with all spines placed on the dendrites. In this latter step, which can take into account the non-linear interaction between a spine and a backpropagating action potential, the parameters were further adjusted in such a way to result in an overall LTP level consistent with the experimental findings shown in Fig 1A. It is important to stress that the key point in this paper was not to explore the parameter space or to find their best values but to study if, how, and to what extent, the proposed scheme was able to take into account the basic experimental findings on BDNF-dependent spike-time-dependent LTP.

As mentioned when discussing Fig 2, we explicitly modeled eighteen independent spines, each containing the mechanisms described above with the parameters reported in Table 2. To introduce the physiological variability of the biochemical pathway dynamics in the model, the α_*pp*_ value in each synapse was drawn from a random uniform distribution. The number of synapses was not important for the scope of the paper. We found it a convenient number to illustrate and demonstrate that the overall effect measured at the soma was the result of a number of independent synapses. The key point here is that, as we will discuss later, the experimental findings cannot be reproduced by modeling a single synapse or a group of identical synapses. It should also be noted that there are many sources of noise that could affect the model behaviour. For example, random background synaptic activity could jitter the interaction between the elicited EPSPs and the bAPs. However, due to the large number of stimuli repetitions and the slow processes that they activate, this contributed to the overall behaviour in a way similar to the random localisation of the spines. The same would be with variability in the morphological and/or electrophysiological spine parameters.

The table shows only the model parameters introduced in this work. All model files and the Python scripts used to run the simulations described in the paper are available for public download under the ModelDB section of the Senselab database (http://senselab.med.yale.edu, a.n. HYPERLINK "http://modeldb.yale.edu/244412" 244412).

In summary, we have introduced a biophysical model of spike timing-dependent LTP at the Schaffer collateral synapse s of hippocampal CA1 pyramidal neurons. The model took explicitly into account, for the first time, several experimental findings on the BDNF-dependent biochemical pathways.

### LTP elicited by a 70x 1:1 t-LTP induction protocol

**Fig 3:**
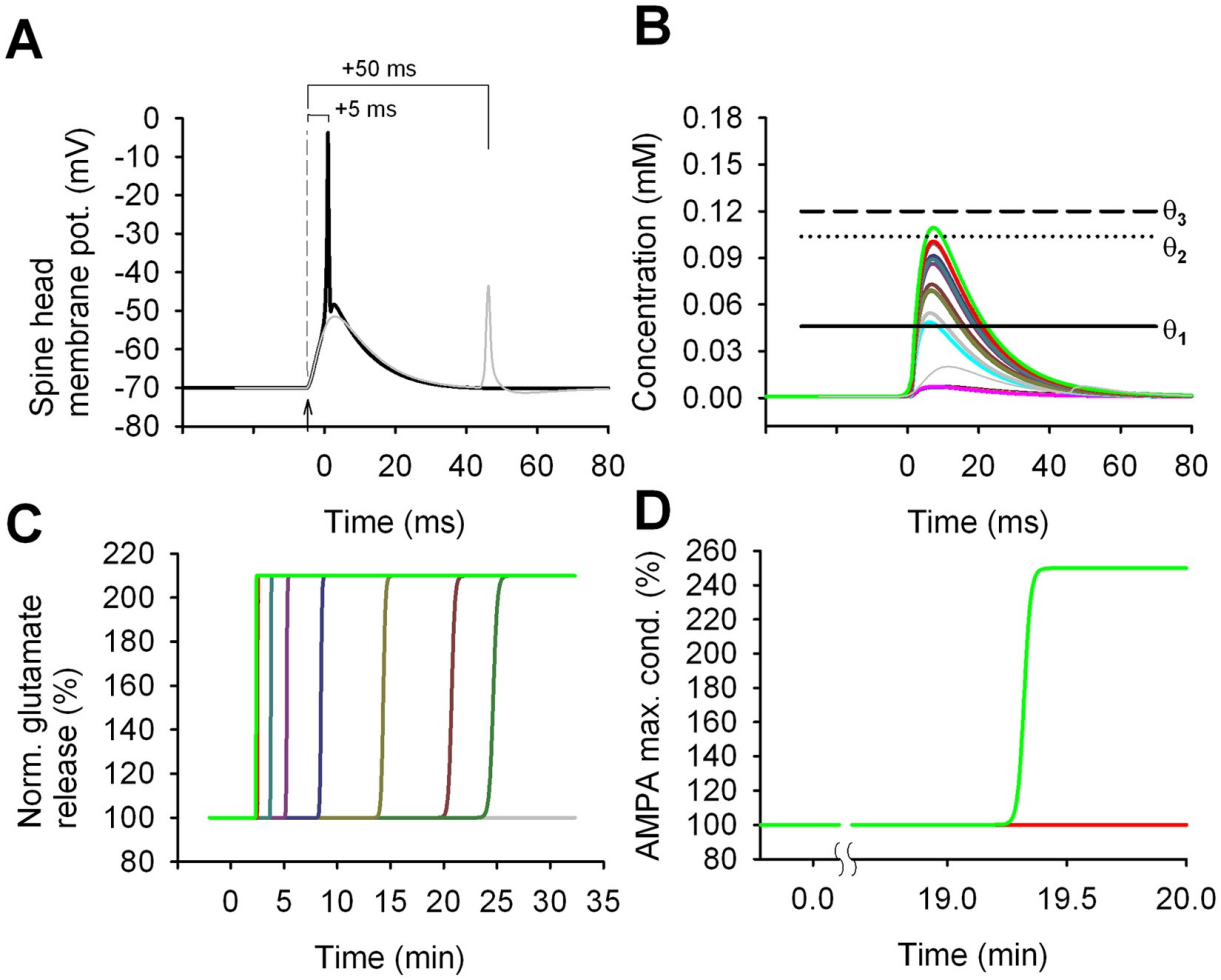
Signals triggered by the 70x 1:1 t-LTP protocol. A) Membrane potential in a spine during a single induction stimulus (black trace); an EPSP was triggered (arrowhead) Δt = 5 ms before the arrival of a bAP (t = 0 was the time of the bAP peak); the grey trace shows the result for Δt = 50 ms. B) The [Ca^2+^]_i_ transient generated in the individual spines by a single conditioning stimulus; the thin grey trace shows the transient in one of the spines for Δt = 50 ms. C) and D) show the time course for the normalized glutamate release and the peak AMPA conductance, respectively, in individual synapses, after the train of 70 conditioning stimuli. Note that the induction protocol triggered pre-synaptic potentiation in almost all synapses; post-synaptic potentiation was induced in the only synapse (green trace) in which the [Ca^2+^]_i_ crossed the threshold θ_2_ during each conditioning stimulus. Note that the vertical axes of panels C and D do not have the same scales.

In Fig 3A, we plotted the membrane potential at a spine head during a conditioning stimulus in which a synaptic activation (arrow) was paired with a bAP elicited with a Δt = +5 or +50 *ms*. The same time course was typically observed at all synapses. Note that for Δt = +50 *ms* (Fig 3A, thin grey trace) the synaptic activation and the bAP could be considered as completely separate events. In this case, the maximum voltage deflection observed in the spine head was approximately 22 mV during the EPSP alone and 17 mV for the bAP. With a Δt = +5 *ms*, the two events overlapped and summed nonlinearly, with a maximum deflection of 66 mV. The nonlinear summation of an EPSP paired with a properly timed bAP has been experimentally observed [40], and in our model was a key factor in inducing LTP. It can be explained by considering that the depolarization caused by the synaptic activation has the effect of inactivating the K_A_ channels, allowing a bAP arriving within a relatively narrow time window to better propagate in the dendrite and the spine. The resulting depolarization released the NMDA receptor Mg-block and allowed a supralinear Ca^2+^ influx.

The [Ca^2+^]_i_ time course, recorded in the 12 spines distributed along one of the oblique dendritic branches during a synchronous activation of all synapses, is shown in Fig 3B (colored lines correspond to different spines). For comparison, we also plotted [Ca^2+^]_i_ in a single spine for Δt = +50 *ms* (grey trace). In all 12 spines of the dendritic segment shown at higher magnification in Fig 2B, the [Ca^2+^]_i_ transiently raised above the θ_1_ threshold for a Δt = +5 *ms*, whereas none of the spines in the other branch (compare blue bracket in Fig 2B) reached the θ_1_ threshold (remaining coloured transients in Fig 3B). The [Ca^2+^]_i_ transient was significantly different among spines. This occurred because the back-propagation of an AP depends on the local dendritic properties. Since the *RM* release is proportional to the amount of [Ca^2+^]_i_ above the θ_1_ threshold, the spines with larger [Ca^2+^]_i_ transients (e.g. bright green and red traces in Fig 3B) were able to accumulate in a shorter time the amount of *RM* required to activate the pre-synaptic mechanisms of plasticity. This resulted in t-LTP induction (in terms of an increase in the glutamate release) earlier than in other spines (Fig 3C dark green and brown traces). Spines for which there was a higher release of RM were potentiated earlier and with a faster transition (bright green trace); spines with lower RM release switched to a potentiated state later and with a slower transition (e.g. dark green trace). Two spines did not release a sufficient amount of RM to trigger potentiation (Fig 3B cyan and thick grey). In only one spine the [Ca^2+^]_i_ transients crossed also the θ_2_ threshold triggering the postsynaptic potentiation mechanisms with a time course depending on TrkB activation (Fig 3D bright green trace).

In agreement with the experiments (Fig 1A), it took around 25 *min* after the induction protocol to switch all synapses to a potentiated state. In the model, we assumed that this could be caused by the slow time constants of the biochemical pathways involved with the retrograde messenger (see *α*_pp_ in Table 2). As expected, since the 1:1 t-LTP protocol was in general not able to generate enough Ca^2+^ entry to cross the θ_2_ threshold, the AMPA conductance, which was modulated by the post-synaptic plasticity mechanisms, did not increase for all but one of the synapses (Fig 3D).

Taken together these results suggest that, in order to be consistent with the experimental findings, it was necessary to make the physiologically reasonable assumption that the *RM*-dependent mechanisms needed to generate a different response at each synaptic location, which is a physiologically plausible condition. This was an important issue that is usually not considered in implementing subcellular models for synaptic transmission. Alternatively, it is possible that long time constants in downstream processes (not explicitly modeled here), such as the incorporation of new glutamate-containing vesicles into the readily releasable pool, are responsible for the approximately 25 min delay in completing the induction of synaptic potentiation [41,42].

### LTP elicited by a 25x 1:4 t-LTP induction protocol

**Fig 4:**
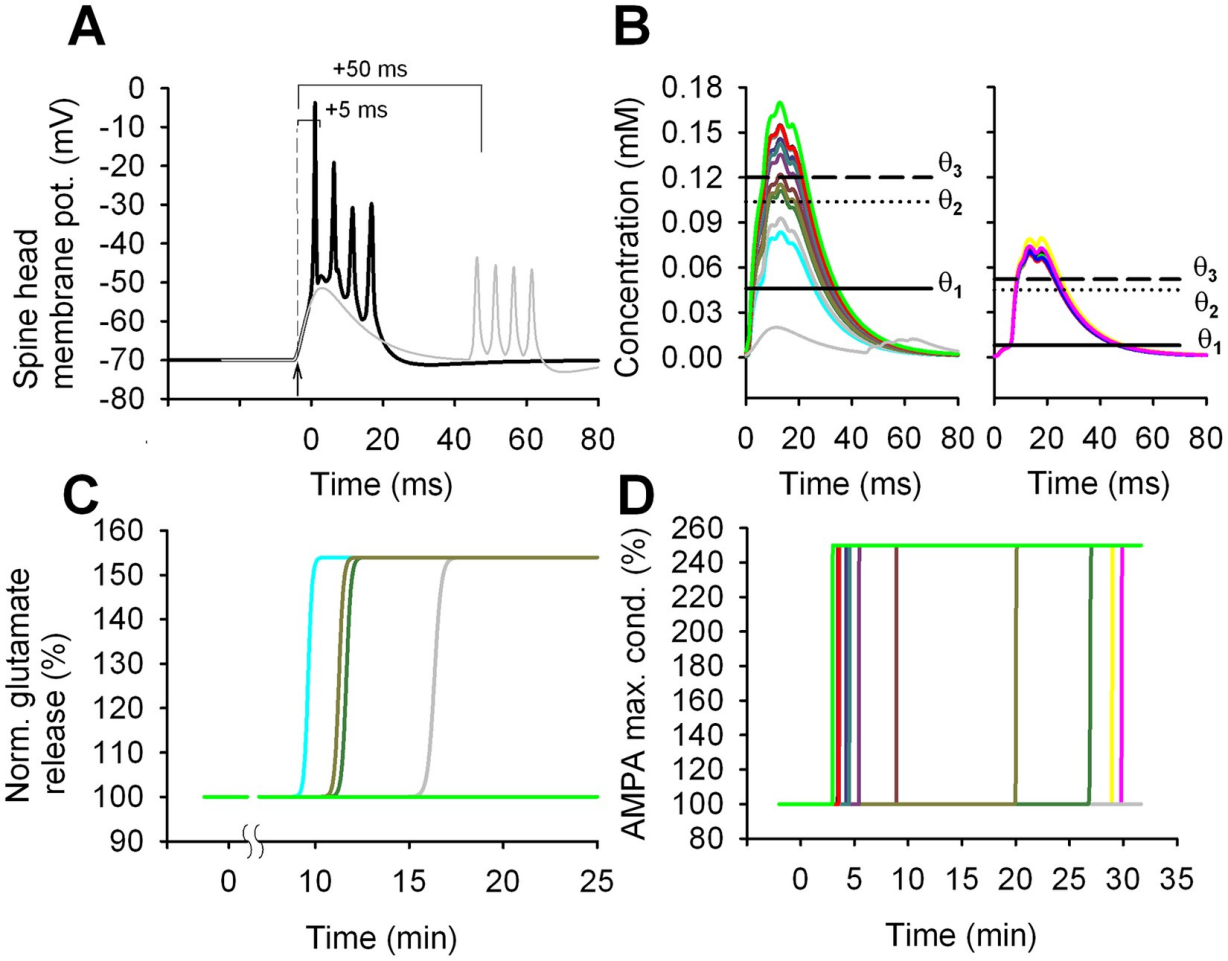
Signals triggered by the 25x 1:4 t-LTP protocol. A) Membrane potential in a spine during a single induction stimulus (black trace); an EPSPs was triggered (arrowhead) before, Δt = 5 ms, the arrival of four bAPs (t = 0 is the time of the first bAP peak); the grey trace shows the result for Δt = 50 ms. B) The [Ca^2+^]_i_ transient generated in all the 18 spines by a single conditioning stimulus; the grey trace shows the transient in one of the spines for Δt = 50 ms; for clarity, the two sets of spines (compare Fig 2B) are shown in two separate panels. C) and D) show the time course for the normalized glutamate release and the peak AMPA conductance, respectively, in individual spines, after the train of conditioning stimuli. Note that the induction protocol triggered pre-synaptic potentiation only in a few synapses (panel C), whereas the peak AMPA conductance was increased (resulting in post-synaptic potentiation) in most synapses. Note that the vertical axes of panels C and D do not have the same scales.

Pairing one synaptic stimulation with four bAPs (Fig 4A) resulted in [Ca^2+^]_i_ transients spanning a range covering all thresholds (Fig 4B both panels). For 2 of the 12 spines in one branch, the [Ca^2+^]_i_ transient crossed only the θ_1_ threshold, resulting in a pre-synaptic t-LTP induction (Fig 4C, cyan and thick grey traces). In other 2 synapses (Fig 4B left panel, light brown and dark green traces) it was θ_2_<[Ca^2+^]_i_<θ_3_. This indicated the activation of both pre- and post-synaptic mechanisms. For the other 10 synapses, the [Ca^2+^]_i_ crossed also the θ_3_ threshold, eliciting the activation of all the post-synaptic (but not presynaptic) pathways, with a consequent long-term potentiation of the AMPA peak conductance (Fig 4D). For the 6 spines in the other dendritic branch, [Ca^2+^]_i_ crossed the θ_3_ threshold in all spines, but only two spines were potentiated (Fig 4D, yellow and magenta traces), while the other 4 spines were not because they did not release and accumulate enough BDNF in the cleft to activate the downstream signaling cascade. This behaviour allowed us to point out a suggestion of our model that will turn out to be extremely important later: BDNF release was necessary but not sufficient to trigger postsynaptic t-LTP. The model suggested that BDNF must accumulate in the synaptic cleft up to an amount sufficient to activate TrkB receptors, i.e. the release must be sufficiently frequent and strong. During the 25 stimulus repetitions, this condition was achieved for only two of the 6 spines (Fig 4D, yellow and magenta traces).

These findings show how the effect of a 25x 1:4 t-LTP conditioning, as observed from the soma, could result from a complex dendritic signal integration process independently occurring in each synapse, and involving diverse biochemical pathways that may interplay in different ways.

### Average 1:1 and 1:4 t-LTP observed from the soma

**Fig 5:**
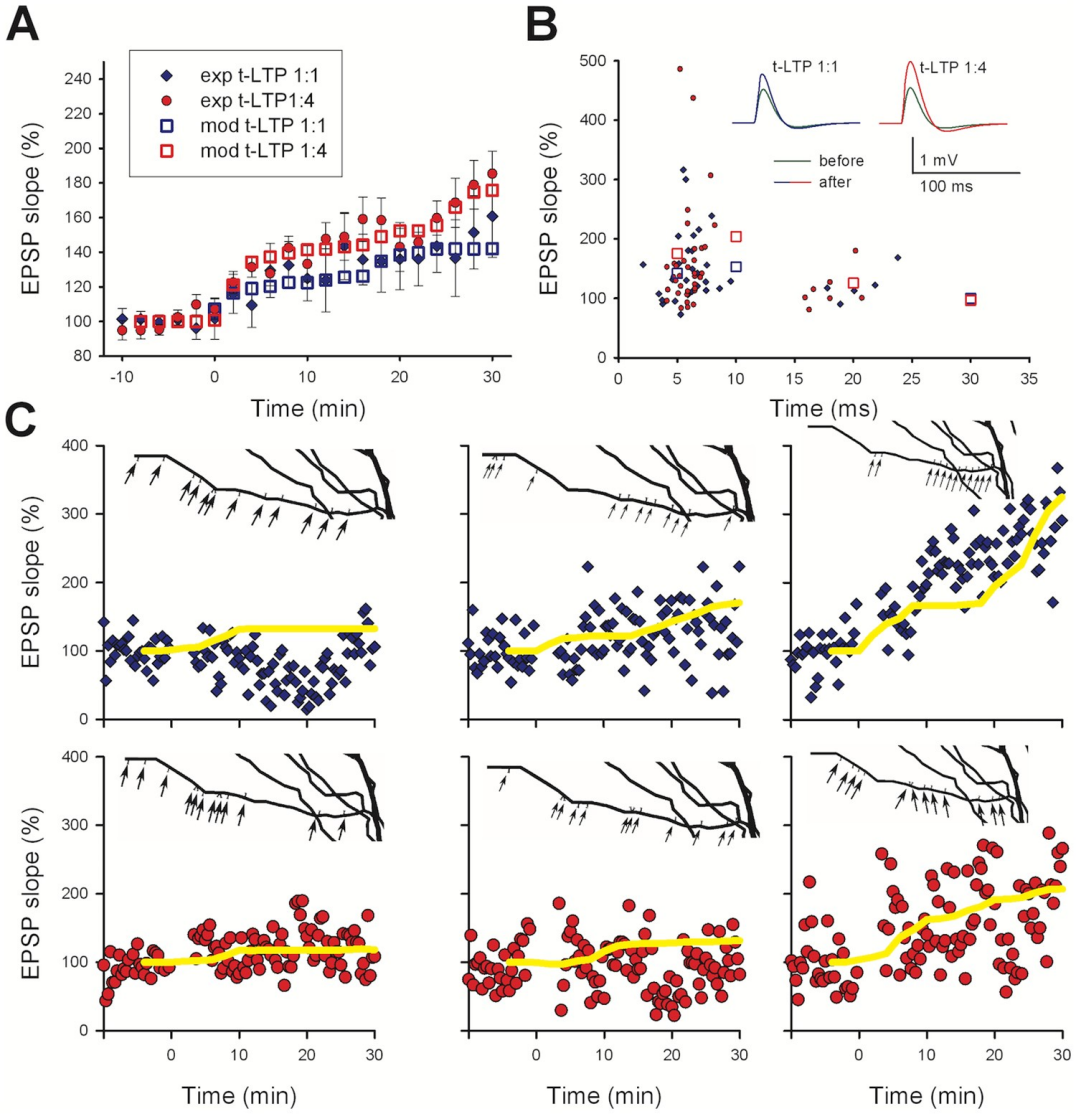
Comparison between model and experiments. A) Average EPSP slope, calculated from the 18 model synapses (red squares, using a Δt = 5 ms) and experimental results after 70x 1:1 t-LTP (blue diamonds with error bars) or 25x 1:4 T-LTP (red circles with error bars) conditioning protocol. B) The final slope of simulated EPSPs (squares) for Δt = 5 ms, 10 ms, 20 ms, and 30 ms are compared with experimental findings (circles). The inset shows typical model traces of EPSPs measured at the soma before (green) and after 1:1 t-LTP (blue) or 1:4 t-LTP (red). C) Experimental findings from individual neurons during 1:1 t-LTP (blue) or 1:4 t-LTP (red) compared with modeling results (yellow traces). In each panel, the inset shows the individual spines along the dendritic branch. The model parameters *α*_AMPA_ and *α*_RMpU_ were set to the following values to fit the experimental data. In the upper panels *α*_RMpU_ = 0.9, 2.2, 7 (from left to right), in the lower panels *α*_AMPA_ = 0.1, 0.5, 1.5 (from left to right).

The results described above were discussed in terms of the processes occurring at the single spine level. We now turn our attention to the average results obtained from several cells. In preliminary simulations, we found that synaptic location and biochemical dynamics in individual spines may result in quite different temporal profiles for the observed t-LTP. This suggests that the overall time course was the result of t-LTP induction and expression mechanisms at each synapse, which respond to the same conditioning protocol by switching to a potentiated state at different times. During a manual trial and error procedure, we thus found a possible combination of synaptic potentiation times that best represented the average experimental results (Fig 5A, compare circles with open squares). The model results for the average EPSP slope measured at the soma were in the range of the experimental data for different values of Δt (see Fig 5B, red and blue squares). The model was also able to reproduce several experimental recordings obtained from individual cells, as shown in Fig 5C for three examples using the 1:1 or 1:4 protocol. For these cases, we found that in order to match the recordings from an individual cell, it was sufficient to distribute the spines along the branch and assume different values for α_USE_ and α_*gampa*_. This is physiologically plausible since these parameters represent the amount of presynaptic glutamate released in the cleft and the density of postsynaptic AMPA receptors, respectively. These factors and the location of activated spines along the dendrite can be expected to be quite different among cells.

These results show that the set of pathways included in the model is able to take into account the main mechanisms underlying BDNF-dependent t-LTP in hippocampal CA1 pyramidal neurons. The model can, therefore, be conveniently used to investigate additional BDNF-dependent effects at these synapses.

### Changes in BDNF release

**Fig 6:**
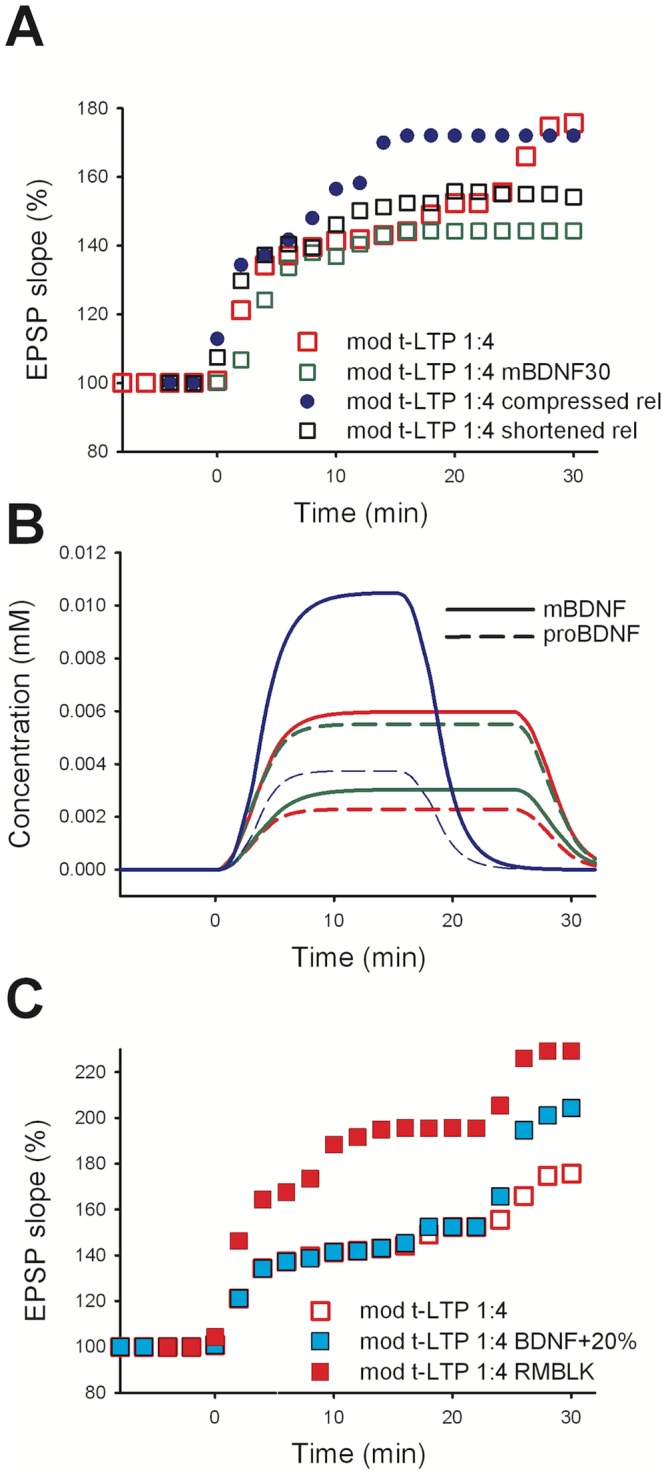
Model robustness and predictions. EPSP slope after STDP induction under different conditions. A) *open red squares*: model control (same model data as in Fig 5 *open red squares*); *open green squares*: model, with the mBDNF:proBDNF ratio set to 30:70 (instead of 70:30 in control); *open black squares*: BDNF release reduced to 15 min; *closed blue circles*: BDNF release compressed to 15 min. B) Solid lines show the concentration in mM of mBDNF over time, dashed lines show the proBDNF. Color coding as in panel A. C) Removal of the RM block (RMBLK in the legend; *closed red squares*) resulted in 1:4 t-LTP protocol inducing both pre- and post-synaptic t-LTP, with a 50% increase in the overall potentiation. Increasing by 20% the concentration of BDNF inside the vesicles (*closed cyan squares*) resulted in a 25% increase of LTP.

To test the behavior of the model under different conditions of BDNF release, we selected two physiologically plausible cases related to specific biochemical processes that may be of particular interest. As mentioned before, the proportion of mBDNF and proBDNF inside the BDNF-containing vesicles is currently unknown, and it may be an important factor in regulating the series of biochemical cascades that they activate. Based on indirect measurements [38,43], in our control model we had set this proportion to 70:30 (see the *postsynaptic mechanisms* section). To test what would happen if this ratio was changed for physiological or pathological reasons, we carried out a set of simulations using the 25x 1:4 t-LTP protocol using a 30:70 proportion for mBDNF:proBDNF. The simulation results are summarized in Fig 6A (green squares). As expected, lowering the initial amount of mBDNF (Fig 6B from control, solid red line, to mBDNF30, solid green line) resulted in a weaker t-LTP in the first minutes after induction (see open green squares in Fig 6A up to 6 min). In Fig 6B we show that in this case the mBDNF level was partially restored to the level observed with a 70:30 ratio (compare the red dashed line with the solid green line) by the cleaving action of PC on the higher level of proBDNF. The lower concentration of mBDNF yielded a higher latency in the expression of potentiation (see green squares in Fig 6A in the range of 8–16 min after induction of t-LTP) and an overall t-LTP after 30 min that was approximately 50% less pronounced than in control. This may appear surprising since it could be argued that the overall amount of BDNF released was the same as in control. This effect can be explained by the lower overall mBDNF accumulation during the 25 min period after conditioning, caused by extrasynaptic diffusion and reuptake processes. The delay in the accumulation of mBDNF resulted in a lower total concentration in the cleft at 25 min from induction failing to potentiate the spines with least [Ca^2+^]_i_ influx. The final result was a failure to potentiate those synapses in which the BDNF accumulation was not enough to activate TrkB receptors and trigger the potentiation of the relative AMPA conductance. It should be stressed that here we were interested in illustrating the possible consequences of changing the mBDNF:proBDNF ratio before they were released from the post-synaptic vesicles. An additional effect, not included in this model, would be caused by the two factors preferentially binding, after their release, to different receptors (proBDNF to p75 receptors versus mBDNF binding TrkB receptors) followed by receptor mediated endocytosis (discussed e.g. in [3]).

Another process that we investigated was the duration for the vesicular release of BDNF. In contrast with the extremely fast glutamatergic mechanisms of release, the processes underlying BDNF release are known to last for minutes ([11,26,29]; reviewed e.g. in [20]). To be consistent with experimental findings, under control conditions we used a 30 min time window, but what would be the consequence of having the release reduced or compressed into a shorter time window? For example, reducing the release to a 15 min window corresponds to a proportionally reduced BDNF level; the model predicted that this would result in a 40% reduction in t-LTP expression, with respect to control (Fig 6A, open black squares). Instead, compressing (i.e. releasing the same amount of BDNF during a shorter time interval) the entire release process leads to an overall faster dynamics, through which the same amount of BDNF was released within a shorter time; under this condition the model predicted that, although the final amount of t-LTP would be the same as control (Fig 6A, blue circles), the maximum induction would be reached much earlier. The results obtained with the model were rather robust against changes in starting parameters (see Fig A in S1 Text), and suggesting that the overall time course of t-LTP recorded at the soma could reflect the compound effect of the dynamics of the release process at the individual synaptic contacts.

### Experimentally testable predictions

The kinetic scheme of the model was constructed to include a small set of building blocks needed to take into account the constraints that can be derived from experimental findings on the biochemical pathways that may be involved in BDNF-dependent t-LTP. We considered it a useful template to explore the consequences of selective changes in one or more of the pathways, in the attempt to elaborate a few model predictions fostering future experimental work. We were particularly interested in figuring out the possible mechanisms that could be exploited to rescue normal cognitive functions that under pathological conditions reduce synaptic transmission, such as Alzheimer’s disease (AD). For example, we could assume that under AD conditions AMPA/NMDA receptors were less efficient in providing the depolarization needed to activate enough Ca^2+^ entry and to activate LTP induction [44–47]. This could occur, for example, because of AD-dependent alterations in the process of binding glutamate to receptors or in a conformational change affecting their peak conductance [44]. A possible rescue mechanism, in this case, could be a minimal perturbation of the pathways, in such a way to increase the magnitude of LTP that could be induced by the STDP protocol.

Experimental findings suggest two possible ways in which this could be obtained. The first way is related to the clear separation in the induction processes between the pre- and post-synaptic t-LTP pathways. It has been shown that both processes did not occlude each other [11]. In the model, we have obtained this condition by including in the kinetic scheme a block of retrograde messenger production for high levels of [Ca^2+^]_i_. By removing this block, one could obtain a further increase in potentiation (with a 1:4 protocol), since the two pathways would both be activated. In Fig 6C, we showed the results after removing the “*RM block*” from the kinetic scheme (see Fig 2A). Under this condition, the combined pre- and post-synaptic t-LTP expression during a 1:4 protocol would be substantially higher than in control, with an overall 50% increase (Fig 6C closed red squares). It should be stressed that, to the best of our knowledge, this was the only explanation for the surprising experimental finding that a 1:4 t-LTP protocol does not activate a presynaptic component that is instead induced by a 1:1 t-LTP protocol.

A second possibility to increase the LTP level during pathological conditions may be to increase BDNF release. There are clear experimental indications suggesting that BDNF expression, and therefore most likely also BDNF release, is increased in response to physical exercise ([48–53]; for a recent review see [54]). A direct connection between exercise, elevated brain BDNF levels, and rescue of synaptic function in Alzheimer’s disease has been described recently [55]. Also, a compensatory elevation of BDNF in AD affected brain areas has been reported (reviewed e.g. in [56,57]), indicating that BDNF-dependent compensation of AD related synaptic deficits might be a spontaneously occurring endogenous protective mechanism that exists even in the absence of physical exercise—but that is further exploited—by physical exercise.

Next, a set of simulations was then carried out with the BDNF release being increased in all spines by 20% (Fig 6C, closed cyan squares). Also under these conditions, the model predicted an increase of the overall potentiation, which in this case was 25% higher than control. However, it should be noted that this increase would strongly depend on the number of spines that were not potentiated by the BDNF released under control conditions. An increase in BDNF release would thus recruit also those synapses and could result in an increase of the overall potentiation observed at the soma. A specific increase of BDNF release, without affecting other processes, has been previously demonstrated experimentally [11].

Taken together, the model predictions might contribute to design experimental investigations aiming to enhance LTP induction in order to rescue neuronal pathologies underlying learning deficits under pathological conditions.

## Discussion

BDNF signaling drives a widespread arsenal of synaptic functional and structural plasticity processes that shape synaptic circuits in many regions of the mammalian brain. Failure of this signaling pathway is known to take part in the progressive development of pathological neurodegenerative diseases such as AD [58] and Huntington's disease [59,60] with loss of memory formation and recall. To begin to sort out the role and intricacies of the many biochemical pathways involved in these processes, a detailed kinetic model of the BDNF signaling driving LTP would be very useful. The main aim of this study was thus the construction of such a model, making available to the scientific community a starting modular representation, able to capture most of the current knowledge on BDNF-driven t-LTP. The minimal set of signaling pathways implemented here, constrained by specific experimental findings [11], include a cascade of reactions that can be individually subjected to further additions/extensions to take into account more specific molecular interactions. It can be argued that the process of releasing BDNF that will act on the same compartment where the release occurred is rather peculiar. However, it should be noted that this type of autocrine signaling is often observed for hormones and other growth factors. For BDNF, this is discussed in [3], and most recent experimental evidence is demonstrated in [11,13,61,62].

The proposed kinetic scheme is a useful framework that can be extended in future work to explore in more details additional mechanisms that are known to be involved in synaptic plasticity, such as Arc protein-dependent processes [63], the role of proBDNF/p75 signaling in t-LTD, or CAMKII auto-phosphorylation to account for synaptic bi-stability [19] and its role in both learning and forgetting [64–65].

The model provides an explicit biophysically and physiologically plausible representation, at the subcellular level, of the interplay among the timing of evoked pre- and post-synaptic activity, the active properties of the membrane, and the intracellular Ca^2+^ dynamics. The study of the conditions under which a local Ca^2+^ influx can trigger the induction of t-LTP in Schaffer collateral to CA1 neurons, allowed us to suggest the requirements for BDNF-dependent signaling at individual synaptic contacts. The results obtained in this work go beyond a simple reproduction of the main experimental results on the BDNF-dependent processes underlying t-LTP. We were able to make experimentally testable predictions on how and to what extent it would be possible to affect the overall magnitude of t-LTP, by manipulating specific synaptic transmission pathways. In this respect, we explored three possible alterations:

1. remove the block for the retrograde messenger released under high [Ca^2+^]_i_ level condition; this would add a presynaptic contribution to t-LTP during the 1:4 t-LTP protocol. In order to act on this mechanism, the retrograde messenger responsible for carrying the induction signal to the presynaptic terminal, and the associated biochemical pathways, need to be identified first. The currently available experimental data seem to rule out NO or endocannabinoids as RM in this respect.
2. increase the mBDNF/proBDNF ratio in single vesicles; the rationale for testing this hypothesis is that there is indirect experimental evidence suggesting a large range for this ratio inside the vesicles. In principle, a larger proportion of mBDNF could generate more t-LTP. In practice, however, a ratio too high would interfere with the magnitude of t-LTD induction, which depends on proBDNF/p75 signaling [1,8,9], with potentially important but unpredictable effects on cognitive functions. One possible manipulation that might reduce such unpredictable effects could be to increase the mBDNF content selectively in vesicles with initially low mBDNF/proBDNF ratio (e.g. by changing PC activity), also taking into account that the mBDNF/proBDNF ratio is known to depend on the stimulus repetition frequency [36]. However, although proBDNF/p75 signaling in conventional LTD is clearly established [66–68], its role in t-LTD is currently unknown and should be addressed in future studies.
3. increase the overall amount of BDNF released upon stimulation. This is by far the most interesting prediction, as it could have direct implications for anti-depressive therapies, known to restore cortical BDNF/TrkB signaling [69] and can possibly also counteract dementia [70]. In the model, the effect of increased BDNF release is based on the physiologically plausible hypothesis that not all of the stimulated synapses are actually potentiated by a conditioning stimulation. This can occur for a variety of reasons, such as dendritic location, and physiological variability of the local biochemical or electrophysiological conditions. The effect may be even more important during any given normal *in vivo* activity, rather than under the more tightly controlled *in vitro* conditions. Our model suggests that the non-potentiated synapses could be recruited by increasing the BDNF release, leading to an increased magnitude of t-LTP. This can be obtained by either pharmacological or external factors. For example, it has been shown that the overall expression of BDNF can be significantly increased by physical exercise [54] suggesting that more BDNF containing vesicles are available for release under these conditions. Apart from optogenetic and transcranial magnetic stimulation techniques to be designed for selective increase of BDNF release, the effect of increasing BDNF release could be experimentally tested *in vitro* by applying ultrasound stimulation to hippocampal brain slices, which has been shown to increase the amount of endogenously secreted BDNF [71] by more than 20%.

Another important result of the model is the clear evidence that, what is observed with electrophysiological recording at the soma, cannot be reproduced from scratch by modeling a single synaptic contact and/or a single point neuron. We consider this a key point for both modelers and experimentalists: modelers, interested in implementing biologically accurate rules for synaptic transmission, plasticity, and dendritic signal integration, must take into account the physiological variability of the biochemical processes, independently occurring at individual synapses according to the local electrophysiological and biochemical environment [72]; experimentalists, interested in extracting as much as possible information from somatic recordings, will better understand the large scatter in the time course of STDP induced synaptic changes at individual synaptic contacts, and could eventually use the model to deduce location and biochemical properties of the potentiated synapses from the STDP time course.

The mechanisms we combined to reproduce the cascade of gating activations could configure a highly unstable dynamical system. In order to demonstrate that our model is robust against small changes of the parameters, we performed 5 new simulation sets, each of which including two simulations where a specific model parameter was increased or decreased by a small fraction. These parameters were chosen because they control the most critical points of the dynamical system. For both the BDNF- and the RM-mediated plasticity chain, we have chosen to vary the [Ca^2+^] threshold that controls the activation of the mechanism and the gain factor that controls the amount of induced potentiation. In our model, these parameters control the beginning and the end of the chain of reactions leading to synaptic plasticity. Therefore, if the model would introduce instability at any point of its cascade of reactions, this would become evident after changing the [Ca^2+^] threshold and not after changing the final gain factor. In contrast, if instability was present in the system, but it was due to non-linear interaction of the potentiated synapses, the unstable behavior would be triggered by changes in either of the two parameters. For the RM-mediated plasticity chain, we selected an additional parameter in the middle of the chain. This controls the RM production and makes it relevant to distinguish between pre- or post-synaptic location of the potentially instable mechanism. The modeling results are shown in the Fig A in S1 Text. The panels A and B show the effects of parameter perturbations in the 25x 1:4 t-LTP-driven, BDNF-mediated plasticity chain. It is evident that a 20% change of the gain factor α_AMPA_ (Eq 16) effective at the end of the activation chain yields an effect similar to a 10% change in the [Ca^2+^] threshold θ_2_ (Eqs 8 and 9), which affects the entire cascade of gating activations. The panels C and D show results for the same kind of test for the RM mediated 70x 1:1 T-plascticity chain RM-mediated. Also in this case, a parametric perturbation at the beginning (θ_1_ Eq 4), in the middle (θ_RM_ Eq 5), and at the end (α_RMpU_ Eq 7) of the cascade of mechanisms yielded similar effects. These results indicate that the model is loosely sensitive to small perturbations of its key parameter, the [Ca^2+^] threshold. In the model construction, we used a sharp transition for the [Ca^2+^] threshold θ_1_ having set σ_1_ = 0.01e-3 mM to simplify the model tuning. In order to show that this very specific choice is irrelevant with respect to our results, we performed one supplemental simulation using a 100 times smoother transition (σ_1_ = 0.001 mM = θ_1_/46, Eq 4). However, this simulation did not disclose any changes in the model behavior.

One limitation of the model is the lack of LTD pathways in the kinetic scheme. The reason for this choice is the current lack of sufficiently detailed experimental constraints on the BDNF-dependent pathways that could be involved in the induction of LTD. The model can be considered a relatively simple template that can be extended to include LTD mechanisms, as soon as more experimental data become available. It should be noted that a synaptic stimulation after postsynaptic spikes (a classic induction protocol for spike-time-dependent LTD) will not generate enough Ca^2+^ entry to activate the cascade of processes leading to t-LTP (Fig B in S1 Text).

Finally, this model provides a foundation to investigate the multi-scale link between the short t-LTP induction period (in the seconds range) and those cases in which plastic changes occur at a much longer time-scale (several minutes), such as BDNF-dependent STDP. The delay in BDNF release may encode for crossing a threshold of relevance caused by a stimulation that shall be remembered for many seconds. For example, this delay can take into account the time of arrival of rewards expected in procedural learning and mediated by dopamine signals. So far there is experimental evidence for BDNF vesicular release lasting at least 5 min [11,29]. The release of BDNF beyond this limit, if confirmed by experimental recordings, opens up the possibility to further fine-tune, on a longer time scale, the extent of how much of the initial learning stimulus is finally converted into a long-lasting memory. These processes might involve additional biochemical cascades regulating neuromodulatory transmitter signaling such as dopamine, noradrenaline or acetylcholine pathways [72].

The overall organization of these mechanisms can thus provide, for example, the neural correlate for the synaptic eligibility traces expected in reinforcement learning to solve the distal reward problem [73]. The model presented here can be used to investigate this type of problem, first at the subcellular level in a single cell and then to extract an effective and computationally more efficient algorithm to be used in large-scale network simulations, where the use of the full implementation would require prohibitively long computing times.

## Methods

### Experimental methods and data

STDP experiments were performed on transversal hippocampal slices (350- to 400-μm thickness) from either P15-P23 Wistar rats (Charles River, Sulzfeld, both sexes), or P25-P35-day-old male BDNF+/- mice bred on a C57Bl/6J genetic background [74] or their WT littermates, respectively, as described previously (compare [11] and references therein). All electrophysiological experimental data were taken from [11]. In short, timing-dependent (t-)LTP was induced with repeated pairings of one presynaptically induced EPSP, evoked by stimulation of Schaffer collaterals and one, two, or four postsynaptic APs induced by somatic current injection (2–3 ms, 1 nA) via the recording electrode in current clamp configuration. T-LTP was induced after a 10 min baseline recording. Cells were held in the current clamp mode at -70 mV. Pairings were repeated 20–150 times depending on the specific protocol. T-LTP was induced by pre-post pairings (indicated by positive spike timings) consisting of either 1 EPSP/1 AP stimulation (70–100 repeats at 0.5 Hz), 1 EPSP/2 AP stimulation (50 repeats at 0.5 Hz), or 1 EPSP/4 AP stimulation (20–35 repeats at 0.5 Hz). Whole-cell recordings were performed at 30.5 °C ± 0.2 °C, with pipettes (pipette resistance 6–10 MΩ) filled with internal solution containing (in mM): 115 potassium gluconate, 10 HEPES, 20 KCl, 4 Mg-ATP, 0.3 Na-GTP, 10 Na-phosphocreatine, 0.00075 CaCl_2_; pH was adjusted to 7.4 using KOH (280–290 mosmol/kg). The bath solution contained (in mM): 125 NaCl, 2.5 KCl, 25 NaHCO_3_, 0.8 mM NaH_2_PO_4_, 20 glucose, 2 CaCl_2_, 1 MgCl_2_, saturated with 95% O2 and 5% CO2 (pH 7.4; 304–306 mosmol/kg). Whole-cell recordings were obtained using either an EPC8 patch clamp amplifier connected to a LiH8+8 interface or an EPC10 patch clamp amplifier (HEKA, Germany) operated with PATCHMASTER software (HEKA, Germany). Data were filtered at 3 kHz and digitized at 10 kHz. Data analysis was performed using FITMASTER (HEKA, Germany) and Mini Analysis software (Synaptosoft, USA). Data analysis was performed as described previously [11]. Matched unpaired controls (negative controls) were performed for quality control with ongoing stimulation of 45min but in absence of any t-LTP induction paradigm [11]. Every recording of t-LTP started with 10 min baseline recording of EPSP at 0.05 Hz. EPSP slopes were calculated from the initial 2 ms after EPSP onset. The mean slope of this baseline was set to 100%. After the 10 min of baseline recording (time interval from -10 to 0 min in all graphs) the STDP protocol was executed, and all subsequent EPSP slopes were normalized to the 100% value during baseline recording.

To describe the experimental results, we extensively use the terms induction and expression to refer to two distinct phases of t-LTP; LTP induction is the pattern of electrical activity in pre- and postsynaptic neurons that triggers the second messenger processes (e.g. intracellular Ca2+ or cAMP elevation) that set in motion the biochemical processes underlying enhanced synaptic transmission; LTP expression is the biochemical mechanism that accounts for the altered synaptic strength at the potentiated synapse, e.g. incorporation of new postsynaptic AMPA receptors, increased probability of presynaptic transmitter release, spine growth.

### Computational methods

All simulations were implemented using the NEURON simulation environment (v7.4, [75]) using the variable time step feature. Additional custom code was written in Python. Model files will be available for public download under the ModelDB section of the Senselab database (http://senselab.med.yale.edu, accession number 244412). For all simulations, we started from a pre-existing CA1 pyramidal cell model [30], downloaded from Senselab (accession number 55035). In this model, already validated against a number of different experimental findings achieved in rat CA1 neurons [30], voltage-gated sodium (*I*_*Na*_) and delayed rectifier potassium (*K*_*DR*_) conductances were uniformly distributed throughout the dendrites, while the *K*_*A*_ [30] and *I*_*h*_ (hyperpolarization induced inward current) conductance linearly increased up to 500 μm from the soma. For the purposes of this paper, the peak K_*A*_ conductance was increased by 30%, with respect to its original value to account for the shorter dendrite of CA1 neurons in mice compared to rats.

To take into account local dendritic integration processes and allow for some physiological variability in the subcellular mechanisms underlying plasticity at individual synapses, eighteen explicit dendritic spines were modeled. The differences among spines are described in *Results*. Each spine was implemented with two compartments, one for the spine neck and one for the spine head. Following experimental indications [76], the spine neck compartment was 2 μm long with a diameter of 0.5 μm, and the spine head was 0.264 μm long and 1 μm thick.

Active ion channels were inserted in the spine head and included L-, N-, and T-type Ca^2+^ ion channels and Ca^2+^-dependent K channels downloaded from a previously published model ([77], ModelDB acc.n. 151126). The resting Ca^2+^ concentration was set at 50 nM and, consistently with experimental evidences for the spine specific Ca^2+^ dynamics [76], the spine intracellular [Ca^2+^] extrusion pump and buffering mechanisms were approximated with a single exponential decay (τ_Ca_ = 12 ms [76]).

### AMPA and NMDA receptor models

AMPA and NMDA receptor channels were placed on the spine head. Their kinetic models were adapted from [78] to include the vesicular cycling dynamics of glutamate release [31]. The effects of glutamate reuptake and diffusion away from the synaptic cleft were modeled using a fast exponential decay (τ_glu_ = 0.1 ms) for the extracellular glutamate concentration [79,80]. With respect to the original model [78], we did not use the slow adapting and [Ca^2+^]_i_-dependent component of the AMPA kinetic, which in the original paper take into account a special form of BCM like synaptic meta-plasticity rule. Also, in our simulations the intracellular and extracellular concentration of Na^+^ and K^+^ ions was fixed.

### Model stimulation protocols

For the purposes of this work, we considered the following stimulation protocols delivered at 0.5Hz:

***1*:*1 t-LTP***: 70–100 repetitions of a single pre and post-synaptic stimuli, for a total stimulation time of 140–200 sec.
***1*:*2 t-LTP***: 50 repetitions of one pre- and two post-synaptic stimuli (at 200Hz), for a total stimulation time of 100 sec.
***1*:*4 t-LTP***: 25–30 repetitions of one pre- and four post-synaptic stimuli (at 200Hz), for a total stimulation time of 50–60 sec.

In all cases, simulations were repeated using different delays, Δt from +5 ms to +30 ms, between the pre- and post-synaptic activation. Post-synaptic action potential activation was obtained with a suprathreshold somatic current pulse (1 nA for 2.5 ms). Test stimuli were delivered at 0.05 Hz before and after the induction protocols described above. The EPSP elicited by the last test stimulus delivered before initiation of the t-LTP induction protocol was used to compute the reference (100%) EPSP slope (maximum value of the time derivative in the 2 ms after EPSP onset). All figures show the EPSP slopes computed from the EPSP elicited after t-LTP induction and normalized by the reference slope described above.

## Supporting information

S1 Text Model behaviour sensitivity to parameters and effect of anti-causal stimuli. To demonstrate that our model is robust against a change in parameter values, we ran new simulations, in which a specific model parameter was changed, in most cases, by a ±10% or ±20% fraction. This range was chosen to test if the overall parameter configuration was stable or close to instability. If the model in a stable condition, we do not expect significant changes for any parameter’s change within this relatively small range. We choose to test several parameters controlling the dynamics and the overall amount of LTP, such as the [Ca^2+^] and RM thresholds (*θ*_*1*_, *θ*_*2*_, and θ_RM_ Eqs 4, 5, 8 and 9), the overall gain factors, α_*RMpU*_ (Eq 7) and α_*AMPA*_ (Eq 16), and the steepness of the sigmoid function activating the 1:1 t-LTP pathway, *σ1* (Eq 4). As can be inferred by looking at panel B in Figs 3 and 4, variations in *θ*_*1*_, and θ_2_ may result in a different number of synapses crossing the respective thresholds for LTP induction. In practice, this will result in a roughly proportional change in the overall amount of LTP observed at the soma. The ±10% change investigated here (Figure A, panels B and D) resulted in negligible difference in the overall amount of LTP. A proportional change was also observed by modifying α_*RMpU*_ and α_*AMPA*_ (Figure A, panels A and C). A change in the pathway producing RM was also rather robust after a ±10% change (Figure A, panel E), and the same occurred for a quite drastic 100-fold change in the parameter regulating the steepness of the function activating the 1:1 t-LTP pathway, *σ*1 (Figure A, panel F).
(DOCX)
