# Supplementary material for: A kinetic model for Brain-Derived Neurotrophic Factor mediated spike timing-dependent LTP
Source: PLoS Comput Biol. 2019 Apr 24;15(4):e1006975. doi: 10.1371/journal.pcbi.1006975 (PMC6502438; doi:10.1371/journal.pcbi.1006975)
Supplement: S1 Text — To demonstrate that our model is robust against a change in parameter values, we ran new simulations, in which a specific model parameter was changed, in most cases, by a ±10% or ±20% fraction. This range was chosen to test if the overall parameter configuration was stable or close to instability. If the model in a stable condition, we do not expect significant changes for any parameter’s change within this relatively small range. We choose to test several parameters controlling the dynamics and the overall amount of LTP, such as the [Ca2+] and RM thresholds (θ1, θ2, and θRM Eqs 4, 5, 8 and 9), the overall gain factors, αRMpU (Eq 7) and αAMPA (Eq 16), and the steepness of the sigmoid function activating the 1:1 t-LTP pathway, σ1 (Eq 4). As can be inferred by looking at panel B in Figs 3 and 4, variations in θ1, and θ2 may result in a different number of synapses crossing the respective thresholds for LTP induction. In practice, this will result in a roughly proportional change in the overall amount of LTP observed at the soma. The ±10% change investigated here (Figure A, panels B and D) resulted in negligible difference in the overall amount of LTP. A proportional change was also observed by modifying αRMpU and αAMPA (Figure A, panels A and C). A change in the pathway producing RM was also rather robust after a ±10% change (Figure A, panel E), and the same occurred for a quite drastic 100-fold change in the parameter regulating the steepness of the function activating the 1:1 t-LTP pathway, σ1 (Figure A, panel F). (DOCX) [file pcbi.1006975.s001.docx]

Supporting Information

To demonstrate that our model is robust against a change in parameter values, we ran new simulations, in which a specific model parameter was changed, in most cases, by a ±10% or ±20% fraction. This range was chosen to test if the overall parameter configuration was stable or close to instability. If the model in a stable condition, we do not expect significant changes for any parameter’s change within this relatively small range. We choose to test several parameters controlling the dynamics and the overall amount of LTP, such as the [Ca2+] and RM thresholds (*θ_1_*, *θ_2_*, and θ_RM_ Eqs.4, 5, 8, 9), the overall gain factors, α*_RMpU_* (Eq. 7) and α*_AMPA_* (Eq. 16), and the steepness of the sigmoid function activating the 1:1 t-LTP pathway, σ1 (Eq. 4). As can be inferred by looking at panel B in Figures 3-4, variations in *θ_1_*, and θ_2_ may result in a different number of synapses crossing the respective thresholds for LTP induction. In practice, this will result in a roughly proportional change in the overall amount of LTP observed at the soma. The ±10% change investigated here (Supp. Figure S1, panels B and D) resulted in negligible difference in the overall amount of LTP. A proportional change was also observed by modifying α*_RMpU_* and α*_AMPA_* (Supp. Figure S1, panels A and C). A change in the pathway producing RM was also rather robust after a ±10% change (Supp. Figure S1, panel E), and the same occurred for a quite drastic 100-fold change in the parameter regulating the steepness of the function activating the 1:1 t-LTP pathway, σ1 (Supp. Figure S1, panel F).


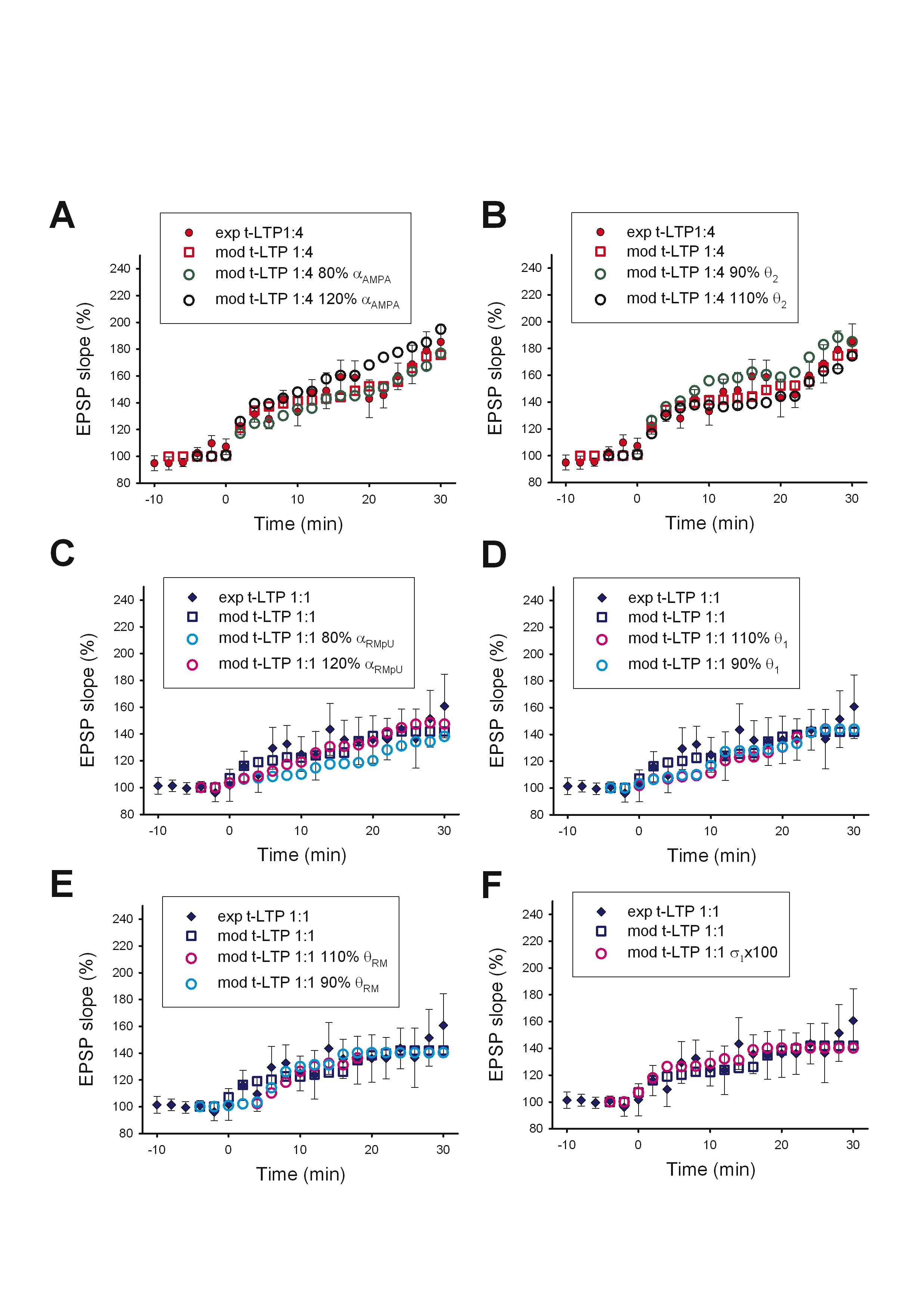


**Supplemental Figure S1. Model behaviour sensi­ti­vi­ty to parameters.** All pa­nels show experimental (closed red or blue cir­cles) and modeling (open red or blue squares) results.

1:4 LTP obtained by: (**A**) a ±20% chan­ge in $\alpha_{AMPA}$, (**B**) a ±10% change in $\theta_{2}$.

The amount of 1:1 LTP obtained by: (**C**) a ±20% change to $\alpha_{RMpU}$, **(D)** a ±10% change in $\theta_{1}$, **(E)** a ±10% change in *θ*_RM_**, (F)** a 100-fold increase of σ1.


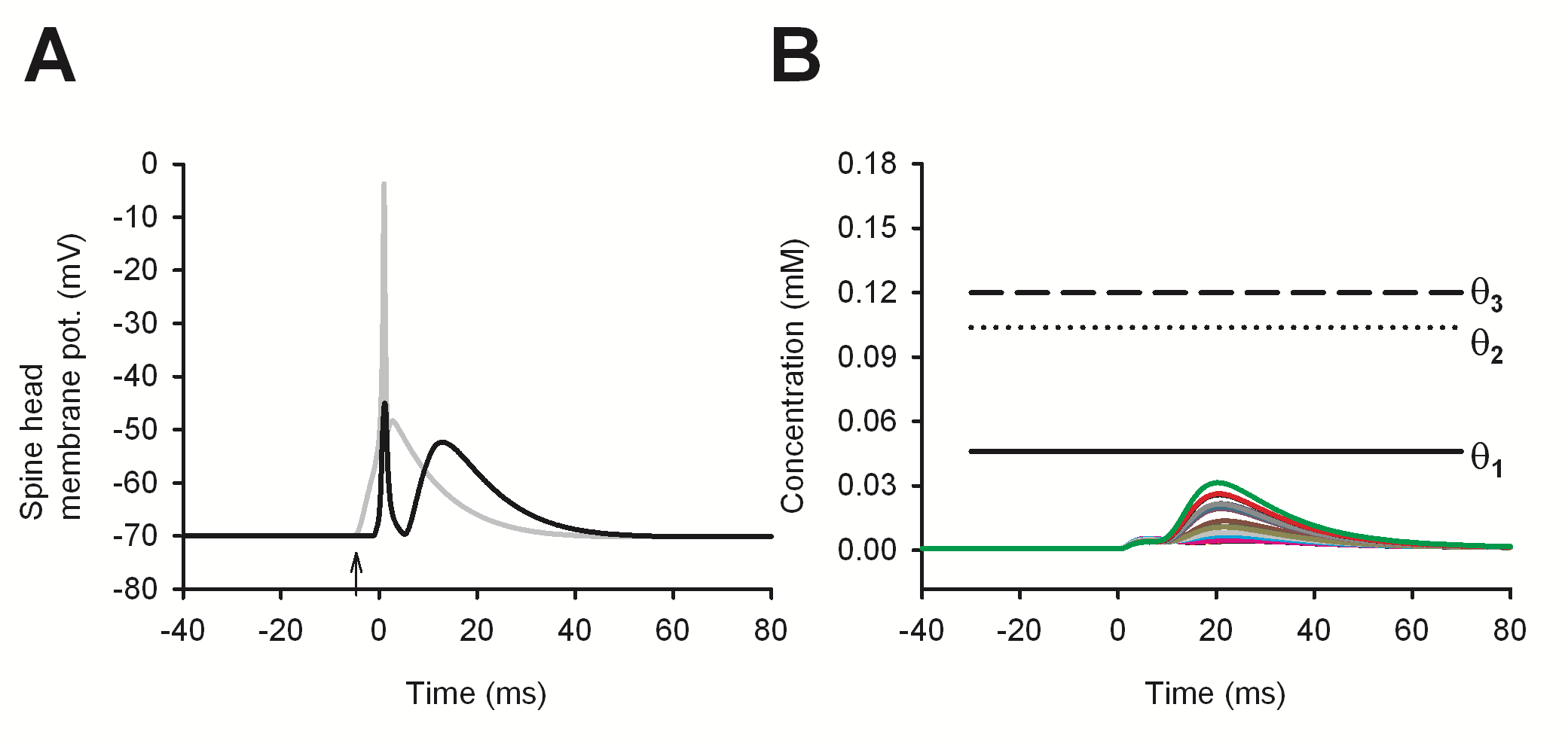


**Supplemental Figure S2. Anti-causal stimuli.** A) Delivery of presynaptic stimulation 5 ms after a bAP invaded the spine head (black trace) failed to further depolarize the EPSP. The grey trace corresponds to the black trace of Fig. 3A. **B)** The anti-causal stimulus failed to trigger a Ca^2+^ influx (grey trace) into the spine head sufficiently high to activate any of the synaptic plasticity mechanisms.

**Sigmoidal Logistic Functions**

The Eqs. 4, 5, 7, 14, 16 use the sigmoidal logistic function to relate two variables through a non linear monotonic function, encoding an ensemble transition from inactive (low) to active (high) states of a population of molecular mechanisms involved in the induction and expression of t-LTP. Our working hypothesis is that the activation of these mechanisms follow a dose-response model with parameters unbounded by the experimental data [1]. This transitions could have been implemented using a Hill function *h(x)=x^n^/(A+x^n^)*, with *n*>1 [2]). The latter can be considered more adherent to the biophysical mechanisms but it implies a higher computational cost (for NEURON running on a PC we verified a 35% difference in CPU time). This occurs because of the internal representation of the algorithms to calculate an *exp* or a *power* on a computer, a sigmoid function (using *exp*), is computationally much more efficient than a Hill function (using *power*). Since we plan to use this model on a large-scale network, we have preferred to implement these curves with a sigmoid function, noting that they can be shown to be mathematically equivalent, as demonstrated below.

Sigmoidal logistic function:

$$f(x)=\frac{1}{1+e^{-x}}$$

Hill function:

$$h(x)=\frac{x^{n}}{A+x^{n}}=\frac{1}{1+\left(B/x\right)^{n}}$$

Where:

$$B=A^{n}$$

Let us change the variable x in the sigmoidal logistic function with:

$$x=\log((y/B)^{n})\equiv e^{-x}=(B/y)^{n}$$

This implies that:

$$x=\log((y/B)^{n})\Rightarrow f(x)=\frac{1}{1+e^{\log((y/B)^{n})}}=\frac{1}{1+(y/B)^{-n}}=\frac{1}{1+(B/y)^{n}}=\mathop{f}\limits^{\sim} (y)$$

# References

1. Thomas N. Hypothesis Testing and Bayesian Estimation using a Sigmoid *E* _max_ Model Applied to Sparse Dose-Response Designs. J Biopharm Stat. 2006;16: 657–677. doi:10.1080/10543400600860469

2. Goutelle S, Maurin M, Rougier F, Barbaut X, Bourguignon L, Ducher M, et al. The Hill equation: a review of its capabilities in pharmacological modelling. Fundam Clin Pharmacol. 2008;22: 633–648. doi:10.1111/j.1472-8206.2008.00633.x
